# Human Infection with Highly Pathogenic Avian Influenza A(H7N9) Virus, China

**DOI:** 10.3201/eid2308.170600

**Published:** 2017-08

**Authors:** Changwen Ke, Chris Ka Pun Mok, Wenfei Zhu, Haibo Zhou, Jianfeng He, Wenda Guan, Jie Wu, Wenjun Song, Dayan Wang, Jiexiong Liu, Qinhan Lin, Daniel Ka Wing Chu, Lei Yang, Nanshan Zhong, Zifeng Yang, Yuelong Shu, Joseph Sriyal Malik Peiris

**Affiliations:** Guangdong Provincial Center for Disease Control and Prevention, Guangzhou, China (C. Ke, J. He, J. Wu);; First Affiliated Hospital of Guangzhou Medical University, State Key Laboratory of Respiratory Disease, Guangzhou (C.K.P. Mok, W. Guan, D.K.W. Chu, N. Zhong, Z. Yang, J.S.M. Peiris);; The University of Hong Kong, Hong Kong, China (C.K.P. Mok, J.S.M. Peiris);; National Institute for Viral Disease Control and Prevention, China CDC, Beijing, China (W. Zhu, D. Wang, L. Yang, Y. Shu);; The Sixth Affiliated Hospital of Guangzhou Medical University, Qingyuan, China (H. Zhou, J. Liu, Q. Lin);; Jinan University, Guangzhou (W. Song);; Macau University of Science and Technology, Macau, China (N. Zhong, Z. Yang)

**Keywords:** influenza, H7N9, HPAI, hemagglutinin, China, highly pathogenic avian influenza, zoonoses, oseltamivir resistance, CMV reactivation, hypoxia, acute respiratory distress syndrome, chickens, poultry, neuraminidase, R292K mutation, viral pneumonia, antimicrobial resistance

## Abstract

The recent increase in zoonotic avian influenza A(H7N9) disease in China is a cause of public health concern. Most of the A(H7N9) viruses previously reported have been of low pathogenicity. We report the fatal case of a patient in China who was infected with an A(H7N9) virus having a polybasic amino acid sequence at its hemagglutinin cleavage site (PEVPKRKRTAR/GL), a sequence suggestive of high pathogenicity in birds. Its neuraminidase also had R292K, an amino acid change known to be associated with neuraminidase inhibitor resistance. Both of these molecular features might have contributed to the patient’s adverse clinical outcome. The patient had a history of exposure to sick and dying poultry, and his close contacts had no evidence of A(H7N9) disease, suggesting human-to-human transmission did not occur. Enhanced surveillance is needed to determine whether this highly pathogenic avian influenza A(H7N9) virus will continue to spread.

Avian influenza A(H7N9) viruses with zoonotic potential emerged in East China in early 2013. From February 2013 through February 20, 2017, a total of 1,222 patients with A(H7N9) disease had been reported to the World Health Organization, with 304 of these patients being reported January 19–February 14, 2017 (*1*,*2*). The overall case fatality ratio was ≈37%. Most patients with A(H7N9) disease primarily had viral pneumonia, with some progressing to acute respiratory distress syndrome (ARDS). Unlike patients with influenza A(H5N1) disease, patients with A(H7N9) disease were more likely to be older and have underlying comorbidities (*3*). In healthy young persons with A(H7N9) infections, symptoms were often mild, and potentially a substantial proportion of these infections was asymptomatic and not recognized (*4*–*6*).

The A(H7N9) virus is a low pathogenicity avian influenza (LPAI) virus that typically causes no signs of disease in birds and has become enzootic in poultry. A(H7N9) infections have been reported in poultry and humans from 22 provinces and municipalities in mainland China, with cases acquired in mainland China sometimes being reported in Hong Kong, China; Macao, China; Taiwan; Malaysia; and Canada (*2*).

Phylogenetic analyses of the A(H7N9) hemagglutinin (HA) genes showed that the viruses that emerged in the Yangtze River Delta region in 2013 rapidly spread to other parts of South and South East China and that distinct virus clades became established in these different geographic regions (*7*). Genetic analysis of A(H7N9) viruses has revealed some adaptations in the virus HA that allow binding to the sialic acid receptors on the epithelium of the mammalian upper respiratory tract. Upon infection in humans, these viruses rapidly acquire mutations in the viral polymerase basic 2 (PB2) gene (*8*). Unlike A(H5N1) viruses, A(H7N9) viruses efficiently infect and replicate in ex vivo cultures of the human bronchus (*9*) and are transmitted between ferrets, albeit inefficiently, by the airborne route (*10*), indicating the substantial potential for efficient human-to-human transmission. Although there have been occasional clusters of cases, some likely caused by limited human-to-human transmission, no evidence of sustained human-to-human transmission has been reported (*2*).

When transmitting among domestic, terrestrial poultry, LPAI type A viruses of the H5 and H7 subtypes can undergo mutations that lead to multiple basic amino acids being present at the HA cleavage site, a signature associated with increased pathogenicity in chickens. Such viruses can disseminate beyond the respiratory and intestinal tracts to affect the brain, liver, spleen, and pancreas and are lethal in chicken; these viruses are then designated highly pathogenic avian influenza (HPAI) (*11*,*12*). Since their emergence in 2013, the A(H7N9) viruses have remained LPAI viruses in poultry, which has made detection and containment of these viruses more challenging than these activities were for A(H5N1), an HPAI virus that usually caused severe disease in poultry flocks.

We report the investigation of illness in a patient in China infected with an A(H7N9) virus with a novel polybasic amino acid sequence at its HA cleavage site, a change that corresponds with increased pathogenicity in poultry and potentially (although not invariably) increased pathogenicity in humans. Relevant epidemiologic information, such as history of contact with poultry and evidence of transmission of infection to close contacts, is provided.

## Methods

### The Patient

The patient was a 56-year-old man with diabetes and hypertension who lived in Lianzhou, China, in Guangdong Province. He raised chickens in his backyard and noticed some of them were sick and dying weeks before his illness. Some of these chickens were slaughtered, cooked, and consumed by the patient and his family members.

**Figure 1:**
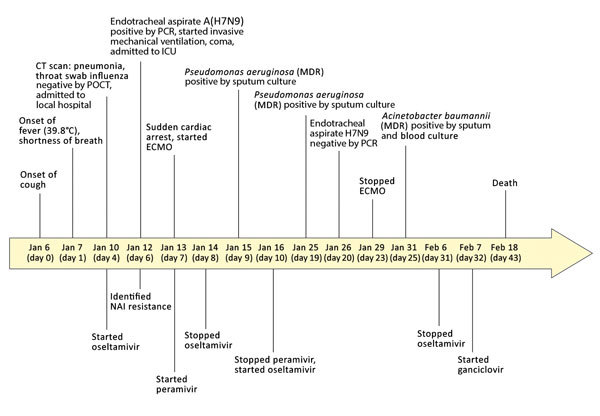
Clinical course of 56-year-old man with diabetes and hypertension infected with highly pathogenic avian influenza A(H7N9) virus, China, 2017. CT, computed tomography; ECMO, extracorporeal membrane oxygenation; ICU, intensive care unit; MDR, multidrug resistant; NAI, neuraminidase inhibitor; POCT, point-of-care test.

On January 7, 2017, day 1 of illness, he had a fever (39.8°C) and cough; shortly thereafter, shortness of breath developed, and on January 10, day 4 of illness, he was admitted to a local hospital (Figure 1). Bilateral pneumonia was diagnosed based on his clinical signs and computed tomography (CT) results. The patient’s throat swab from a point-of-care test was negative for influenza A/B. Because he had a history of contact with poultry, oseltamivir treatment (75 mg 2×/d) was commenced. On day 6, his clinical condition deteriorated markedly, with progression to severe respiratory failure, hypoxia (oxygen saturation 78.6%), and coma. He was transferred to the intensive care unit for invasive mechanical ventilation.

**Figure 2:**
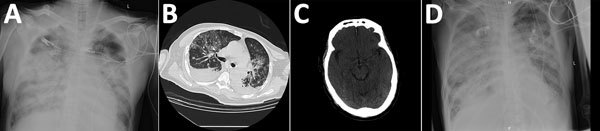
Chest and brain imaging of 56-year-old man infected with highly pathogenic avian influenza A(H7N9) virus, China, 2017: radiograph imaging of chest at day 7 (A) and day 40 (D); computed tomographic scans of the chest (B) and the brain (C) at day 30.

RNA extracted from an endotracheal aspirate acquired on day 6 was positive for influenza A(H7N9) virus. On day 7, he had mild gastrointestinal bleeding and a cardiac arrest. No endoscopy was performed, and he was rescued by cardiopulmonary resuscitation and intravenous injection of norepinephrine. The size and shape of his heart were within reference range. Chest radiography showed he had extensive bilateral infiltration of the lungs with prominent hilar shadowing (Figure 2, panel A). The patient had progressed to severe ARDS. Antiviral therapy was changed from oseltamivir to peramivir (0.6 g intravenous 4×/d), and he was given antibiotics (cefoperazone sodium/sulbactam sodium [2:1], 3 g/8 h). Extracorporeal membrane oxygenation (ECMO) was commenced.

**Figure 3:**
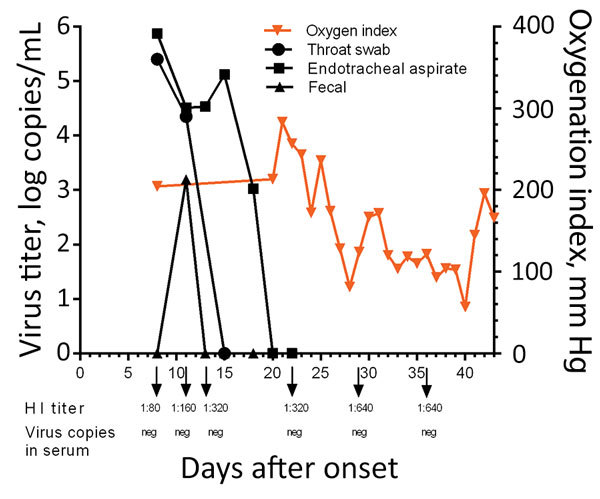
Kinetics of viral load, oxygenation index, and HI antibody titers in 56-year-old man infected with highly pathogenic avian influenza A(H7N9) virus, China, 2017. Arrows indicate the days HI titers and viral titers in serum were acquired. HI, hemagglutination inhibition; neg, negative.

Laboratory tests showed high total leukocyte and neutrophil counts and high levels of procalcitonin, aspartate aminotransferase, serum creatinine, lactate dehydrogenase, blood urea nitrogen, and d-dimers throughout the hospitalization (Technical Appendix Table 1). His hemoglobin level remained low. Continuous renal replacement therapy was applied because of increasing renal dysfunction (days 8–14). His oxygenation index was maintained at around 200 mm Hg by the use of ECMO (Figure 3). On day 10, treatment with peramivir was stopped and oseltamivir recommenced. Viral load in the endotracheal aspirate started to decline on day 16 and was undetectable on day 20 (Figure 3). However, his clinical condition continued to deteriorate gradually after removal from ECMO. Secondary bacterial infections developed with multidrug-resistant *Pseudomonas aeruginosa* (detected in sputum days 9 and 19) and *Acinetobacter baumannii* (detected in blood cultures day 25). Combination antibiotic therapy was used to treat these infections (Technical Appendix Table 2).

Cytomegalovirus (CMV) DNA was detected in patient serum on day 11 of illness, suggesting CMV reactivation. Because of the lack of clinical response to oseltamivir and antibiotics and the detection of CMV DNA in serum, ganciclovir therapy was initiated on day 32 to suppress possible systemic CMV disease. A CT scan of the lungs on day 30 showed multiple bilateral patchy hyperdense lesions and bilateral pleural effusions (Figure 2, panel B). Repeated electrocardiography tracings did not reveal evidence of myocarditis, and Doppler echocardiography examinations showed effective myocardial contraction. CT scan of the brain on the same day showed patchy hypodense lesions with unclear borders in the left corona radiate and right centrum semiovale (Figure 2, panel C), the largest measuring 8 × 10 mm with a CT value (8 H) indicative of lacunar infarction. The CT scan also showed symmetric, bilateral cerebral hemispheres; preservation of gray-white differentiation; no obvious lesions in the brain parenchyma; no deepening or widening of sulci, fissures, and cisterns; no dilatation or deformity in the ventricular system; and no mid-line shift. However, intracerebral edema was evident (Figure 2, panel C).

On day 40, extensive bilateral shadowing of the lung fields with blurring of the costophrenic angles continued to be observed on chest radiography (Figure 2, panel D). On day 43, the patient died.

### Clinical and Epidemiologic Data Collection

The clinical history and epidemiologic information were obtained from the patient at hospital admission and from relatives during interviews. Progression of clinical symptoms and laboratory and radiologic findings were obtained by retrospective chart review.

### Viral Diagnosis

Serially acquired throat swabs, endotracheal aspirates, serum samples, and fecal samples were collected from the patient and stored in viral transport medium. We extracted viral RNA by using QIAamp MinElute Virus Spin kit (QIAGEN, Hilden, Germany) according to the manufacturer’s instructions. The extracted RNA was subjected to reverse transcription and amplification with SuperScript III One-Step RT-PCR System (ThermoFisher, Waltham, MA, USA). We synthesized complementary DNA by using uni-12 primers (5′-AGCAAAAGCAGG-3′), and conducted real-time PCR with an A(H7N9) detection kit (Shanghai Zj Bio-Tech Co., Ltd, Shanghai, China) to detect avian influenza A(H7N9) virus.

### Genome Sequencing and Phylogenetic Analysis

Staff of the Guangdong Provincial Center for Disease Control and Prevention isolated virus from the endotracheal aspirate specimen obtained on day 6 of illness onset. Whole-genome sequencing was implemented on the Ion Torrent S5 platform (ThermoFisher) with a mean read length of ≈200 bp. We analyzed data predominantly with CLC Genomics Workbench 7.5.1 software (https://www.qiagenbioinformatics.com/products/clc-genomics-workbench/). Low-quality reads were trimmed by using CLC trimmer with a quality limit set at 0.05. We assembled filtered reads de novo in CLC under default parameters. Contigs with coverage >10 bp were extracted and blasted against the GISAID (Global Initiative on Sharing All Influenza Data) databases (http://platform.gisaid.org). Sequences with the highest similarity were selected as references for read mapping (parameters: length fraction = 0.8, similarity fraction = 0.8). We obtained influenza A genome sequences by extracting consensus sequences from the mapping results with a coverage depth of >30× at each nucleotide site of the 8 gene segments. The viral sequences generated in this study (Technical Appendix Table 3) were submitted to the GISAID database.

We used the MEGA software version 5.05 (http://www.megasoftware.net) to construct phylogenetic trees, and maximum likelihood trees were constructed with PhyML (http://www.atgc-montpellier.fr/phyml/) by using the general time reversible plus gamma distribution plus proportion of invariable sites model. We estimated node support by the SH-like aLRT method and report values >0.8. Bootstrap values from 1,000 replicates were calculated to assess the reliability of the phylogenetic trees.

### Serology

A(H7N9)-specific antibody titers were quantified in serially acquired serum samples by hemagglutination inhibition (HI) assay by using horse erythrocytes according to the World Health Organization recommended protocol (http://www.who.int/influenza/gisrs_laboratory/cnic_serological_diagnosis_hai_a_h7n9_20131220.pdf). A recombinant A/PR/8/34 virus with the HA and neuraminidase (NA) genes of A/Zhejiang/DTID-ZJU01/2013 (H7N9) was used for serologic tests. All bioassays were conducted in a Biosafety Level 3 laboratory. Seropositivity was defined as an HI titer >1:40.

### Ethical Approval

Guangdong Center for Disease Control and Prevention is legally tasked with data collection on patients in the course of a public health investigation during an emerging infectious disease outbreak. Thus, informed consent was waived.

## Results

This patient initially tested negative for influenza A/B, but on day 6, real-time reverse transcription PCR performed using an endotracheal aspirate showed positive results for A(H7N9) virus RNA. Viral RNA was detectable in the patient’s endotracheal aspirate at high levels (>4 log RNA copies/mL) until day 17 (Figure 3), was undetectable in serum throughout the course of illness, and was transiently detected in the feces on day 11. Antibody to A(H7N9) virus was first detected in serum collected on day 8 (HI titer 1:80); serum titers increased until day 29 (1:640) (Figure 3).

**Figure 4:**
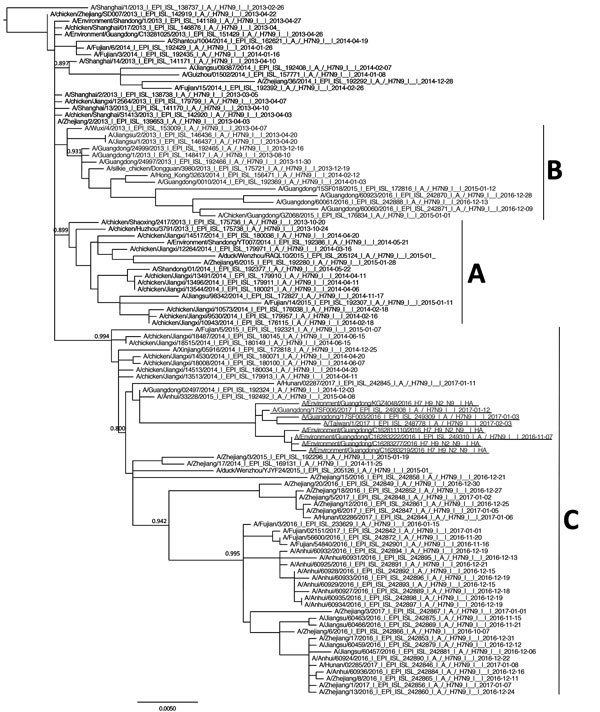
Phylogenetic analysis of the hemagglutinin gene of highly pathogenic avian influenza A(H7N9) viruses in Guangdong Province, China, and reference viruses. Maximum likelihood trees were constructed with PhyML by using the general time reversible plus gamma distribution plus proportion of invariable sites model. Node support was estimated by the SH-like aLRT method, and values >0.8 are shown. Virus clades A, B, and C—previously defined as W2-A, W2-B, and W2-C (*5*)—are labeled. A/Guangdong/17SF006/2017 (from a 56-year-old man), A/Guangdong/17SF003/2016, A/Taiwan/1/2017, and environmental isolates are underlined. Scale bar indicates nucleotide substitutions per site.

We performed phylogenetic analyses on the virus (designated A/Guangdong/17SF006/2017 [17SF006/17]) isolated from the patient on day 6. The HA segment of 17SF006/17 was related to A(H7N9) clade W2-C (*7*) (Figure 4). An insertion that led to the addition of multiple basic amino acid residues (PEVPKRKRTAR/GL) was found at the HA cleavage site, suggesting that this virus might be highly pathogenic in birds (Table) (*13*). A similar mutation was found for 2 other cases of A(H7N9) infection identified in Taiwan (A/Taiwan/1/2017) (*14*) and Guangdong Province (A/Guangdong/17SF003/2016, reported as A/Guangdong/Th008/2017) (*15*). The HA and matrix sequences of these 3 viruses clustered together in our phylogenetic analysis.

**Table:** Key molecular signatures of avian influenza A(H7N9) virus from patient in Guangdong Province, China, compared with other closely related viruses*

Strain	HA				NA		M2		PB2				PA
321–331†	G186V‡	Q226L‡	R292K§	S31N¶	E627K#	K526R**	K702R††	V100A††
A/Guangdong/ 17SF006/2017 (H7N9)‡‡	PEVPKRKRTAR/GL	V	Q		K		N		K	K	K		V
A/Guangdong/ 17SF003/2016 (H7N9)	PEVPKRKRTAR/GL	V	Q		K		N		E	K	R		V
A/Taiwan/1/2017 (H7N9)	PEVPKRKRTAR/GL	V	Q		K		N		K	R	K		A
A/Environment/ Guangdong/ C16283222/2016 (H7N9)	PEVPKGKRTAR/GL	V	Q		R		N		E	R	K		A
A/Fujian/3/2016 (H7N9)	PEIPKG—-R/GL	V	L		R		N		K	R	K		A
A/Zhejiang/3/2017 (H7N9)	PEIPKG—-R/GL	V	L		R		N		K	K	K		V
A/Netherlands/ 219/2003 (H7N7)	PEIPKRRRR/GL	G	Q		R		S		K	R	K		A

*HA, hemagglutinin; NA, neuraminidase; NAI, neuraminidase inhibitor; M2, matrix protein 2; PA, polymerase acidic; PB2, polymerase basic 2. †Cleavage site. ‡Receptor binding site. §Associated with NAI drug resistance. ¶Associated with amantadine resistance. #Associated with increased virulence in mice. **Associated with enhanced viral replication in mammalian hosts. ††Species-associated signature position. ‡‡Virus from patient in this report.

We also sequenced and studied the HA and NA genes of 7 A(H7N9) viruses isolated from environmental samples (from birds at live poultry markets) collected previously in the Guangdong Province. Six of the 7 were mixed with A(H9N2) viruses, and thus, the genes for the internal proteins could not be analyzed. All of the A(H7N9) viruses isolated from the environmental samples had the same HA cleavage site sequence motif as the A(H7N9) human viruses (except for an amino acid change [G] at position −6 from the cleavage site) and clustered with them in the phylogenetic tree, suggesting the emergence of a single subclade of A(H7N9) virus with an HA progressively acquiring HPAI mutations. Similar to other A(H7N9) viruses, the amino acid substitution G180V in the HA protein, which is known to enhance the binding to mammalian α-2,6–linked sialic acid receptors, was found.

The NA genes of 17SF006/17, A/Guangdong/17SF003/2016, and the environmental isolates grouped together, but A/Taiwan/1/2017 did not cluster, suggesting that the origin of this NA gene was distinct (online Technical Appendix Figure, panel A). A mutation associated with oseltamivir resistance (one leading to R292K substitution in NA) was identified in the NA gene of 17SF006/17 virus. The patient had been on oseltamivir for 2 days before the mutation was detected. Both A/Guangdong/17SF003/2016 and A/Taiwan/1/2017 also had the mutation associated with oseltamivir resistance, but the viruses from the Guangdong environmental samples did not. Similar to other A(H7N9) viruses, 17SF006/17 had a mutation leading to the S31N amino acid substitution in the matrix protein, which is associated with amantadine resistance. A mutation in PB2 that causes substitution E627K, which is a key signature of mammalian adaptation, was also found in this virus. The other viral gene segments of the 3 human viruses are not monophyletic and appear to be derived from reassortment with genetically diverse A(H7N9) viruses (Technical Appendix Figure, panels A–G).

To investigate the potential for human-to-human transmission of HPAI A(H7N9), an epidemiologic investigation was conducted immediately after virus detection in the patient. Seventy close contacts (5 family members and 65 healthcare workers) were placed under medical observation for 2 weeks. None of them showed signs of illness.

## Discussion

We report the illness and death of a patient in Guangdong Province who was infected with a putative HPAI A(H7N9) virus carrying an HA cleavage site with multiple basic amino acids. Previous A(H7N9) viruses were low pathogenicity, containing only a single arginine residue at the HA cleavage site. Although the HA cleavage site we describe in this report was not a typical HPAI motif for H7 viruses, similar motifs have been reported in H7 viruses in Chile and Canada that were shown to be highly pathogenic in chickens by intravenous pathogenicity tests (*13*). More studies are needed to determine the pathogenicity of these putative HPAI A(H7N9) viruses (e.g., direct isolation from sick and dying poultry and intravenous pathogenicity index testing in chickens). Viruses isolated from live poultry markets had a sequence motif similar to that of the human isolates (identical except for a glycine at the position −6 from the cleavage site). An additional patient identified in Guangdong Province was infected with an A(H7N9) virus (A/Guangdong/Th005/2017) with HA cleavage site KGKRIAR/GL (*15*). This evidence suggests that the polybasic HA cleavage site was acquired progressively through multiple mutations within the avian species in a subclade of the A(H7N9) viruses.

The patient had reported that chickens in his backyard had started dying in the weeks before his disease onset, indicating the possibility that these chickens were infected with an HPAI and that the patient acquired his infection from them. However, we do not have direct evidence of A(H7N9) infection in these birds, and alternative explanations for their deaths exist.

A(H7N9) viruses having HA cleavage sites with multiple basic amino acids were reported in 2 human cases in the Guangdong Province and Taiwan (Table) (*14*,*15*). The HA sequences of these 2 viruses clustered with that of A/Guangdong/17SF006/2017 in our phylogenetic tree. These viruses also clustered with viruses from the environmental samples from Guangdong, which had a similar sequence motif (1 amino acid difference) at the cleavage site as the human isolates, suggesting the progressive emergence of this HPAI motif in a subclade of A(H7N9) viruses in this region. Although the HA gene segments of these viruses are monophyletic, the other gene segments are not and have diverse origins, suggesting that these HPAI viruses continue to reassort their gene segments. Introduction of the polybasic cleavage site into A(H5N1) was associated with increased viral titers in the respiratory tract, increased virus dissemination to distant organs, increased death in mice and ferrets (but not in macaques), and increased virus replication in endothelial cells (*16*,*17*).

A question of public health interest is whether acquisition of the polybasic cleavage site in A(H7N9) HA enhances pathogenicity of A(H7N9) viruses in humans. Similar to most patients with A(H7N9) disease (*18*), the patient in this report had underlying comorbidities (diabetes and hypertension), which probably contributed to the increased disease severity. The clinical progression of the illness to fulminant viral pneumonia and ARDS was relatively rapid, in spite of commencing oseltamivir on day 4 of illness. The patient required mechanical ventilation and became comatose on day 6 of illness and had a sudden cardiac arrest on day 7, necessitating ECMO. Myocarditis and encephalitic illness were not evident by either clinical observation or imaging investigations, and the most likely cause of the coma was respiratory failure. One of the other patients in Guangdong who had a similar HPAI A(H7N9) virus infection had less severe illness, indicating that the HA cleavage sequence did not invariably increase pathogenicity in humans (*15*).

By day 6 of patient disease onset, 2 days after commencement of oseltamivir, the virus had acquired a mutation leading to an R292K change in the NA protein known to be associated with oseltamivir resistance (*19*,*20*). The other 2 viruses with the same HA cleavage site mutation (A/Guangdong/17SF003/2016 and A/Taiwan/1/2017) also had this oseltamivir resistance mutation. It is likely that the viruses from all 3 patients acquired this resistance mutation after commencement of oseltamivir therapy. Although the emergence of R292K has been reported before in a minority of patients following oseltamivir treatment and treatment failure (*19*), mutations causing this amino acid substitution have not been found in poultry as of May 30, 2017 (*7*), and accordingly, the resistance mutation was not observed in the related environmental samples collected from live poultry markets. The frequency with which R292K has been detected in patients with the polybasic HA cleavage site is a cause for concern. Whether acquisition of the polybasic HA cleavage site in the A(H7N9) virus contributes to the enhanced probability of the virus acquiring the NA R292K during oseltamivir treatment is unknown. Studies of HPAI A(H5N1) viruses in vitro and in vivo have demonstrated that viruses with polybasic residues at the HA cleavage site or the PB2 E627K amino acid change enhances replication efficiency, which might increase the likelihood of resistance emerging under the selective pressure of oseltamivir therapy (*21*–*23*).

NA R292K confers resistance to both oseltamivir and peramivir, the antiviral drugs used in this patient’s therapy (*20*). Viral load in the endotracheal aspirates remained high for 10 days following commencement of antiviral therapy, probably reflecting lack of efficacy of the antiviral regimens. Viral load only began to decline on day 18 of illness, probably because of increasing antibody titers. Thus, the uncontrolled viral replication and emergence of antiviral resistance in this patient possibly contributed to the adverse clinical outcome. In spite of the prolonged infection and high virus titer within the respiratory tract, virus RNA was not detectable in the serum (Figure 3). Thus, systemic dissemination of the virus was unlikely. This patient was not treated with corticosteroids until day 41, shortly before his demise; thus, corticosteroid therapy did not increase viral load or facilitate the emergence of antiviral resistance, a phenomenon speculated to have occurred in other patients in whom oseltamivir resistance mutations arose (*19*).

The emergence of antibiotic-resistant *Acinetobacter* and other bacteria might also have contributed to the adverse clinical outcome. Detection of CMV DNA in serum (rather than in leukocytes) is suggestive of clinically significant CMV viremia following CMV reactivation in a seriously ill patient. CMV viremia is an indication for ganciclovir therapy in immunocompromised patients, which was the rationale for initiating ganciclovir therapy. However, the benefit of using ganciclovir in this context remains unknown.

The patient had increasing levels of serum creatinine and blood urea nitrogen, indicating moderate renal dysfunction, and moderately elevated aspartate aminotransferase, indicating moderate liver dysfunction. Similar features have been previously reported in A(H7N9) patients and are not necessarily indicative of systemic virus dissemination (*8*).

No evidence of human-to-human transmissibility was apparent; family members and healthcare workers who were in contact with the patient did not have evidence of clinical disease. The HA cleavage site mutation that makes avian influenza viruses highly pathogenic in birds does not necessarily affect the transmissibility of the virus between humans. However, unlike LPAI viruses, which are restricted to the chicken respiratory and intestinal tracts, HPAI viruses spread systemically within chickens and are likely to be found at high titer in multiple organs, including muscle. Thus, the risk for zoonotic transmission through handling or butchering infected poultry and consuming undercooked poultry is likely to increase with HPAI viruses.

In summary, we report the clinical disease progression of a patient infected with a mutant A(H7N9) virus that acquired sequence motifs similar to those found in HPAI viruses. The clinical features of human disease with this isolate did not appear to differ from previous infections with low pathogenicity A(H7N9) viruses, and the clinical and virologic evidence suggested that systemic dissemination of the virus did not occur. The emergence of R292K in NA, which is associated with NA inhibitor resistance, probably contributed to the adverse clinical outcome. In China, heightened surveillance of A(H7N9) in humans with severe respiratory disease and poultry is needed to determine how widespread the polybasic HA cleavage sequence has become and to monitor for evidence of oseltamivir resistance.

Technical Appendix Virus isolate information submitted for inclusion into the Global Initiative on Sharing All Influenza Data database, laboratory test results of and clinical treatments for the 56-year-old man infected with the highly pathogenic avian influenza A(H7N9) isolate, and phylogenetic analyses of the remaining 7 segments of A/Guangdong/17SF006/2017.
