# Supplementary material for: Human Infection with Highly Pathogenic Avian Influenza A(H7N9) Virus, China
Source: Emerg Infect Dis. 2017 Aug;23(8):1332–40. doi: 10.3201/eid2308.170600 (PMC5547808; doi:10.3201/eid2308.170600)
Supplement: Technical Appendix — Virus isolate information submitted for inclusion into the Global Initiative on Sharing All Influenza Data database, laboratory test results of and clinical treatments for the 56-year-old man infected with the highly pathogenic avian influenza A(H7N9) isolate, and phylogenetic analyses of the remaining 7 segments of A/Guangdong/17SF006/2017. [file 17-0600-Techapp-s1.pdf]

Article DOI: https://doi.org/10.3201/eid2308.170600

Human Infection with Highly Pathogenic Avian Influenza A(H7N9) Virus, China

Technical Appendix

Technical Appendix Table 1. Laboratory test results of 56-year-old man with diabetes and hypertension infected with highly pathogenic avian influenza A(H7N9) virus, China, 2017*

		Day														
Variable	Reference range	7	8	9	10	11	12	13	14	15	20	22	23	30	38	43
Total white cells, $\times 10^9$ cells/L	3.97–9.15	9.97	8.4	9.93	12.46	17.2	20.9	22.5	22.85	17.9	20.5	10.03	6.65	7.35	21.9	18.8
Neutrophil absolute value, $\times 10^9$cells/L	2–7	8.88	7.1	8.63	10.28	14.3	18.9	18.1	18.36	15.6	19.3	8.47	5.23	5.42	18.8	16.2
Neutrophil ratio, %	50–70	89.1	84.5	86.9	82.5	83.1	90.5	80.7	80.4	86.8	94	84.4	78.6	73.8	85.7	86.3
Eosinophils, $\times 10^9$cells/L	0.02–0.5	0.05	0.01	0.02	0.16	0.19	0.23	0.36	0.25	0.16	0.02	0.25	0.25	0.6	0.2	0.02
Eosinophil ratio, %	0.5–5	0.5	0.1	0.2	1.3	1.1	1.1	1.6	1.1	0.9	0.1	2.5	3.7	8.2	0.9	0.1
Lymphocytes, $\times 10^9$ cells/L	0.8–4.0	0.46	0.75	0.84	1.41	1.93	0.98	2.49	2.76	1.06	0.49	0.82	0.81	0.7	2.04	2.48
Lymphocytopenia		Yes	Yes	No	No	No	No	No	No	No	Yes	Yes	Yes	Yes	No	No
Platelets, $\times 10^9$ cells/L	85–303	291	225	249	213	215	223	194	180	165	84	76	69	168	403	52
Thrombocytopenia		No	No	No	No	No	No	No	No	No	Yes	Yes	Yes	No	No	Yes
Hemoglobin, g/dL	131–172	105	94	106	107	109	107	99	100	108	106	84	91	75	67	85
C-reactive protein, mg/L	0–8.2	165	96.9	ND	ND	ND	103	ND	ND	73.1	244	120.9	103	ND	166	320
Procalcitonin, ng/mL	0–0.10	3.22	18.2	18.5	14.15	10.3	6.85	4.2	2.02	1.05	ND	0.26	0.18	0.14	1.29	56.5
Aspartate aminotransferase, U/L	15–40	143	83	104	111	167	613	405	275	159	393	88	74	121	56	251
Serum creatinine, µmol/L	59–104	150	154	98.4	119.9	117	137	232	173.2	234	145	128.1	119.9	99.5	150	80.8
Lactate dehydrogenase, U/L	120–250	808	631	820	863	890	1,109	876	926	822	811	592	622	482	ND	461
Creatine kinase, U/L	50–310	328	287	480	432	304	183	126	65	40	46	ND	56	46	30	303
Potassium, mmol/L	3.50–5.3	4.36	3.6	4.41	5.44	5.11	4.76	5.15	4.22	3.7	3.6	3.8	3.4	3.95	4.3	3.54
Sodium, mmol/L	137–147	141	145	147	137	135	134	132	140	162	162	162	161	151	137	154
D-dimer, mg/L	0–0.68	50.3	ND	10.8	11.74	8.01	6.28	6.73	ND	7.87	41.6	ND	2.79	9.94	3.78	1.79
Blood urea nitrogen, mmol/L	3.1–8.0	19.6	18.7	11.5	7.52	6.28	6.39	12.5	9.8	15.7	24.1	ND	16.55	16.4	33.9	13

*ND, not determined.

Technical Appendix Table 2. Clinical treatments given to 56-year-old man with diabetes and hypertension infected with highly pathogenic avian influenza A(H7N9) virus

Clinical treatment	Day	Dosage
Mechanical ventilation	Day 6–43	
Extracorporeal membrane oxygenation	Day 7–23, day 40–43	
Continuous renal replacement therapy	Day 8–20, day 38–43	
Antibiotic treatment		
Cefoperazone sodium/sulbactam sodium (2:1)	Day 7–12	3 g, q8h
Imipenem/ cilastatin	Day 12–18	0.5 g, q6h
Linezolid	Day 17–24	0.6 g, q12h
Meropenem	Day 18–23	1 g, q8h
Ciprofloxacin	Day 23–27	0.4 g, q12h
Cefoperazone sodium/sulbactam sodium (2:1)	Day 25–37	3 g, q8h
Amikacin	Day 27–37	400 KU, qd
Prostacyclin	Day 36–38	50 mg, q12h
Prostacyclin	Day 38–43	100 mg, q12h
Cefoperazone sodium/sulbactam sodium (2:1)	Day 38–43	3 g, q6h
Imipenem/cilastatin	Day 43	1 g, q8h
Antiviral treatment		
Oseltamivir	Day 4–7	75 mg, bid
Peramivir	Day 7–10	0.6 g, qd
Oseltamivir	Day 10–31	150 mg, q12h
Ganciclovir	Day 32–43	375 mg, q12h
Antifungal treatment		
Fluconazole	Day 15–27	200 mg, qd
Caspofungin	Day 27–43	50 mg, qd
Steroids		
Hydrocortisone	Day 41	50 mg

Technical Appendix Table 3. Viruses included in this study*

Isolate ID	Isolate name	Subtype	Passage history	Collection date	City
EPI_ISL_249308	A/Guangdong/17SF006/2017	A(H7N9)	E1	2017 Feb 12	Qingyuan
EPI_ISL_249310	A/Environment/Guangdong/C16283222/2016	A(H7N9)	E1	2016 Nov 07	Heyuan
EPI_ISL_249311	A/Environment/Guangdong/KGZ4075/2016	A(H7N9), A(H9N2)	E1	2016 Dec 12	Guangzhou
EPI_ISL_249312	A/Environment/Guangdong/KGZ4068/2016	A(H7N9), A(H9N2)	E1	2016 Dec 12	Guangzhou
EPI_ISL_249313	A/Environment/Guangdong/KGZ4048/2016	A(H7N9), A(H9N2)	E1	2016 Dec 12	Guangzhou
EPI_ISL_249314	A/Environment/Guangdong/C16283277/2016	A(H7N9), A(H9N2)	E1	2016 Dec 07	Heyuan
EPI_ISL_249315	A/Environment/Guangdong/C16283219/2016	A(H7N9), A(H9N2)	E1	2016 Nov 02	Heyuan
EPI_ISL_249316	A/Environment/Guangdong/C162811110/2016	A(H7N9), A(H9N2)	E1	2016 Nov 07	Meizhou

*Information was submitted to the Global Initiative on Sharing All Influenza Data database. ID, identification.

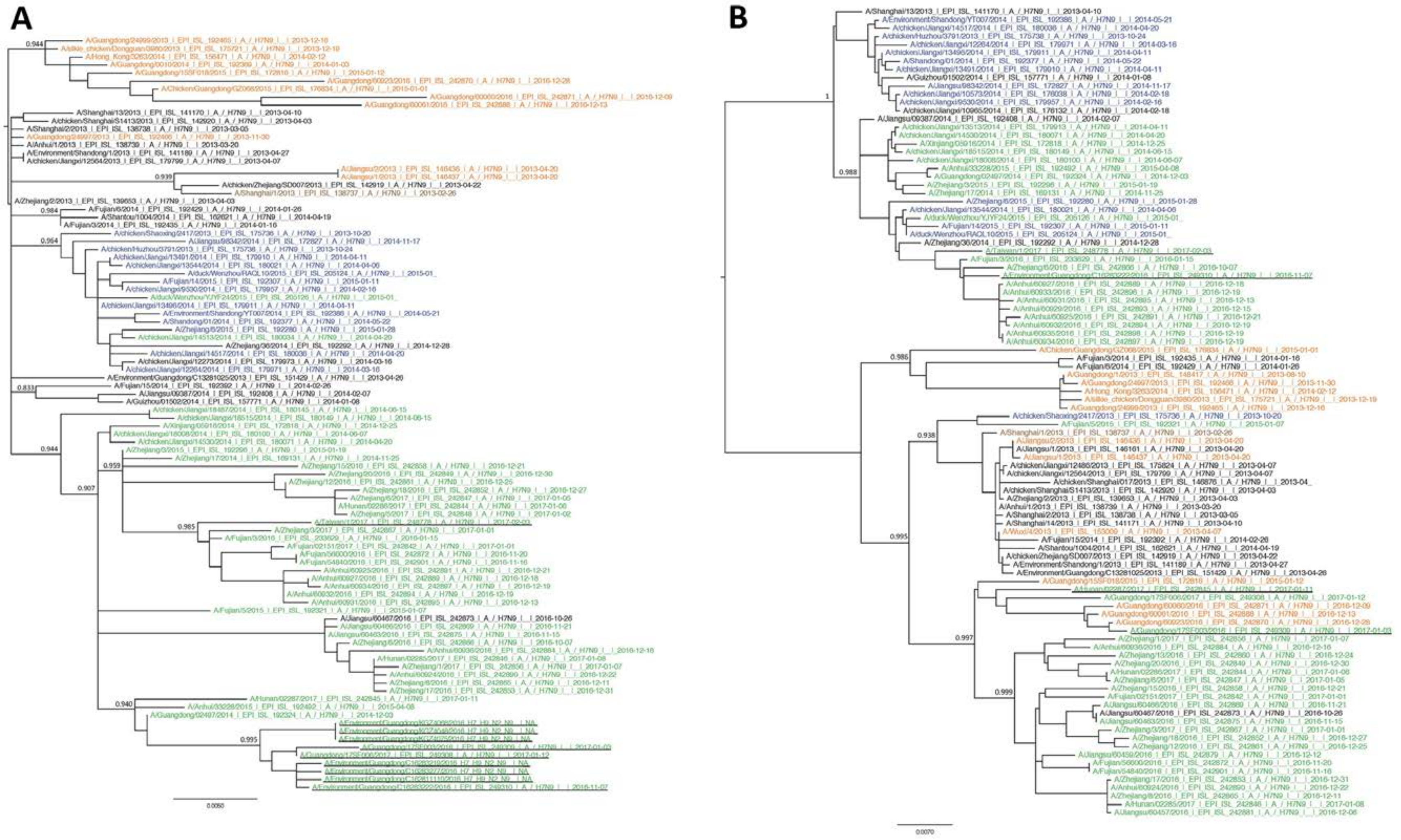
A
B

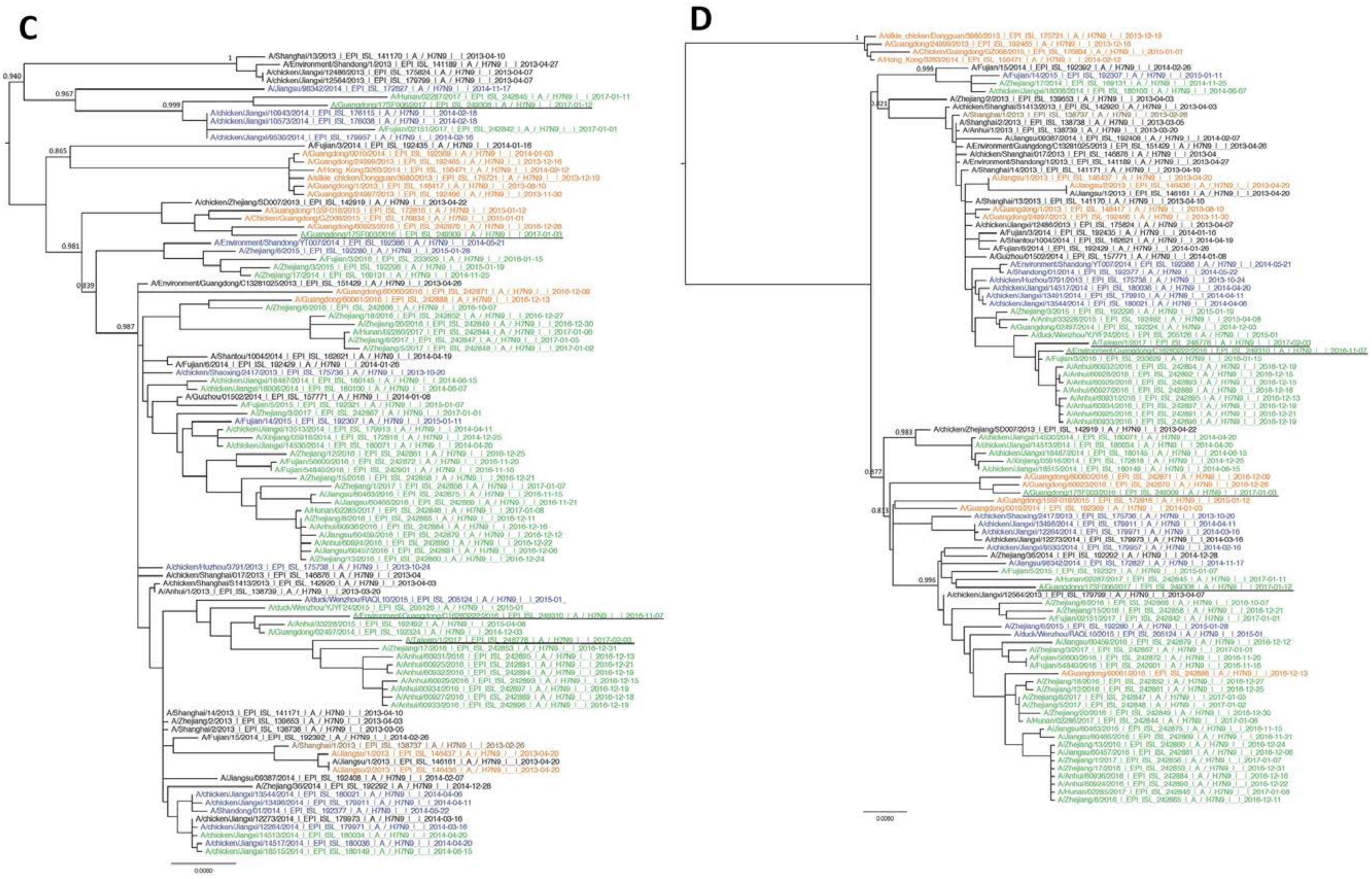
C
D

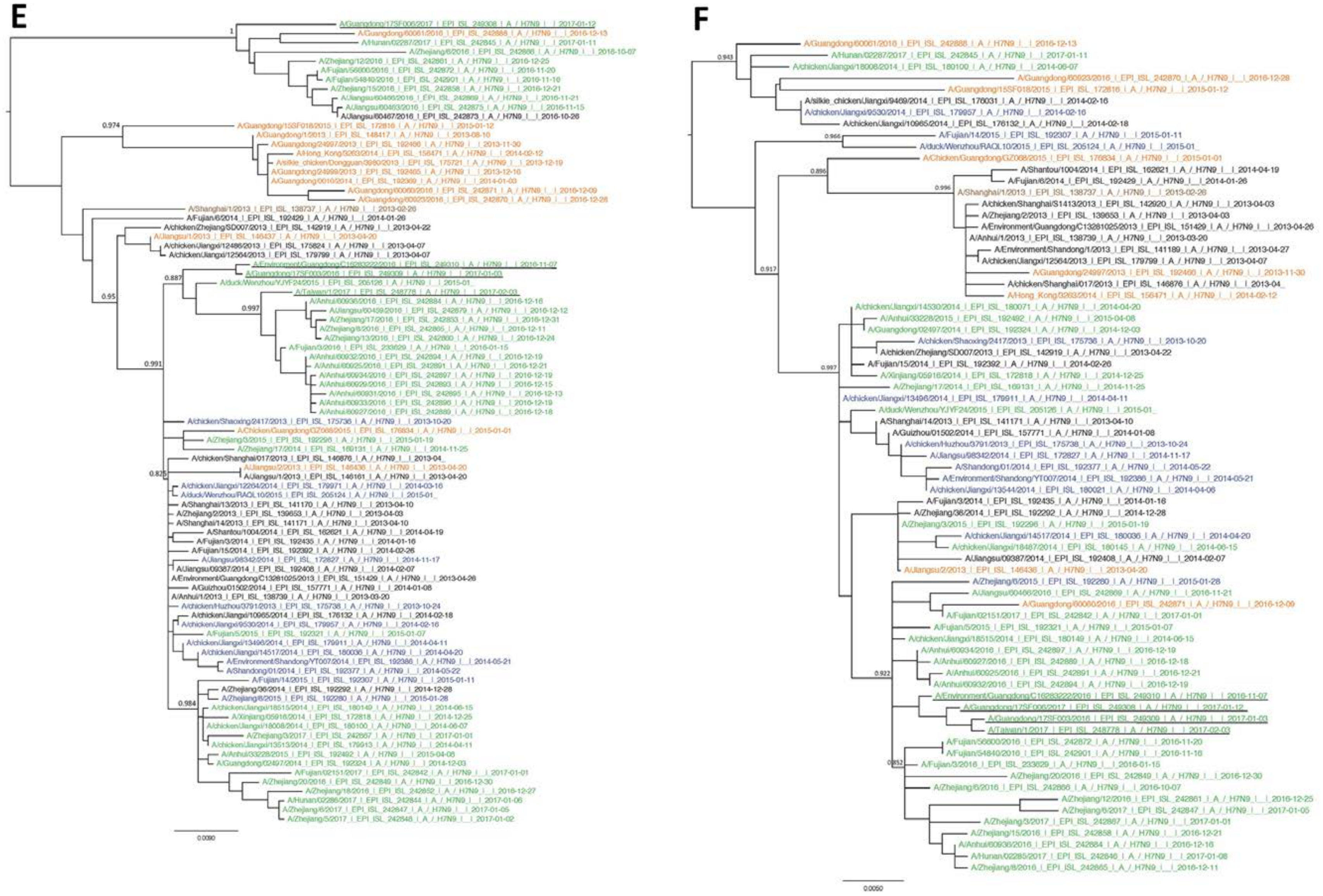
E
F

G

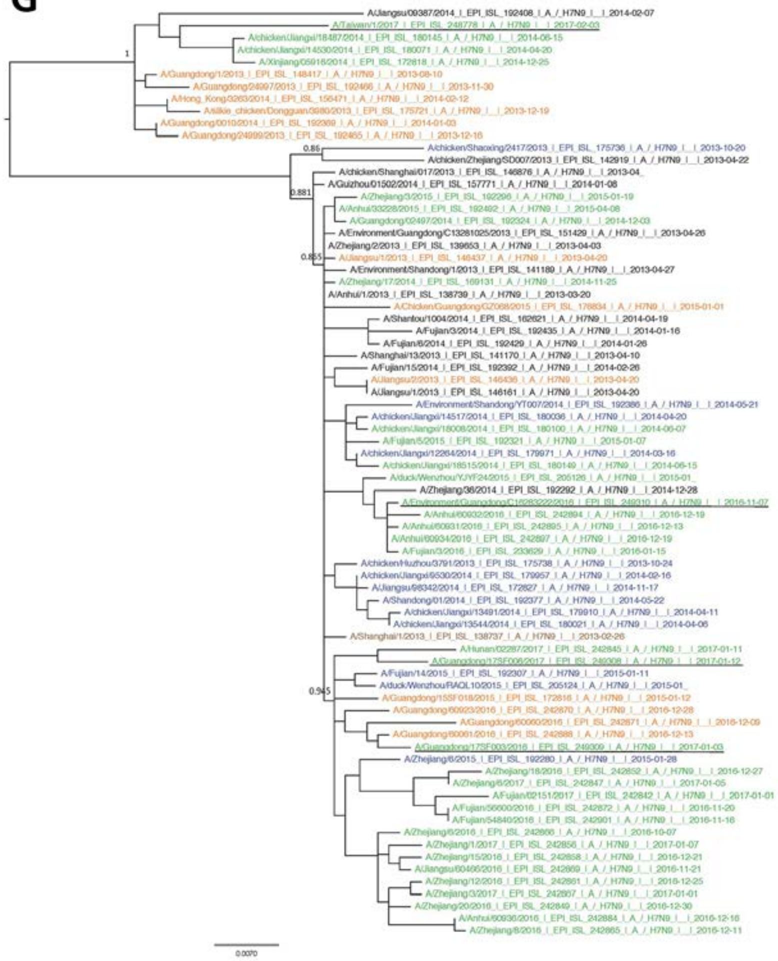

Technical Appendix Figure (following pages). Phylogenetic analyses of the genes of influenza A(H7N9) viruses. Phylogenetic trees of A/Guangdong/17SF006/2017 neuraminidase (A), polymerase basic 2 (B), polymerase basic 1 (C), polymerase acidic (D), nucleoprotein (E), matrix (F), and nonstructural (G) genes. Maximum likelihood trees were constructed with PhyML by using the general time reversible plus gamma

distribution plus proportion of invariable sites model. Node support was estimated by the SH-like aLRT method, and values >0.8 are shown. A/Guangdong/17SF006/2017, A/Guangdong/17SF003/2016, A/Taiwan/1/2017, and the environmental isolates are underlined for clarity. Scale bars indicate nucleotide substitutions per site.
